# Association between cumulative biological risk and hypertension in older adults in Korea: a population-based cross-sectional study

**DOI:** 10.7586/jkbns.26.026

**Published:** 2026-08-26

**Authors:** Hyemin Hwang, Kyeongmin Jang

**Affiliations:** 1 Department of Nursing, Bucheon University, Bucheon, Korea; 2 Department of Nursing, College of Health Sciences, Daejin University, Pocheon, Korea; 3 Research Institute for Community Health Convergence, Daejin University, Pocheon, Korea

**Keywords:** Hypertension, Aged, Biomarkers, Risk factors, Inflammation

## Abstract

**Purpose:**

This study examined the association between cumulative biological risk and hypertension among older adults in Korea using nationally representative data.

**Methods:**

Data were obtained from the 2024 Korea National Health and Nutrition Examination Survey, the most recent dataset released in December 2025. The main analytic sample included 1,551 adults aged 65 years or older. The cumulative biological risk score (CBRS) was calculated using five indicators: anemia, elevated glycated hemoglobin, obesity defined as body mass index ≥ 25 kg/m^2^, elevated high-sensitivity C-reactive protein, and vitamin D deficiency. Hypertension was defined as self-reported physician-diagnosed hypertension. Complex sample analyses incorporated sampling weights, stratification variables, and cluster variables. Complex sample logistic regression was performed using IBM SPSS Statistics version 30.0.

**Results:**

CBRS showed a graded association with hypertension across risk categories. Compared with the low-risk group, the moderate-risk group had higher odds of hypertension (odds ratio [OR] = 1.67, 95% confidence interval [CI] = 1.16–2.40), and the high-risk group had still higher odds (OR = 2.92, 95% CI = 1.87–4.54). Age, monthly alcohol use, and absence of aerobic physical activity were also associated with higher odds of hypertension.

**Conclusion:**

Cumulative biological risk was significantly associated with hypertension among older adults in Korea, suggesting that hypertension in later life may be related to accumulated biological dysregulation. An integrated assessment of multiple biological and metabolic abnormalities may help characterize clustered biological vulnerability and support comprehensive preventive nursing care.

## INTRODUCTION

Hypertension is one of the most common chronic conditions in older adults and remains a major contributor to cardiovascular morbidity and mortality worldwide [1]. In Korea, hypertension imposes a substantial population-level burden. According to the Korea Hypertension Fact Sheet 2024, approximately 30% of Korean adults, corresponding to about 13 million individuals, have hypertension; notably, 5.8 million adults aged 65 years or older are affected by hypertension [2]. Hypertension in later life is not merely a condition of elevated blood pressure but reflects complex age-related biological changes, including vascular aging, arterial stiffness, endothelial dysfunction, chronic low-grade inflammation, and metabolic dysregulation [3]. These biological alterations may interact with obesity, impaired glucose metabolism, inflammatory activation, and micronutrient deficiencies, thereby contributing to impaired blood pressure regulation [4,5].

Previous studies have examined individual biochemical risk factors associated with hypertension, such as obesity, elevated glycated hemoglobin, systemic inflammation, vitamin D deficiency, and magnesium imbalance [6,7]. However, these factors often coexist in older adults and may represent a cumulative biological burden rather than isolated abnormalities. The concept of cumulative biological risk is closely related to allostatic load, which refers to the cumulative physiological “wear and tear” that develops when adaptive responses to repeated or chronic stressors become prolonged or dysregulated [8]. Allostatic load is commonly operationalized by combining biomarkers across multiple biological systems, including metabolic, inflammatory, cardiovascular, neuroendocrine, and immune pathways, to reflect the overall burden of biological dysregulation [8,9]. In this framework, cumulative biological risk is not limited to a single abnormal laboratory value but represents the accumulation of dysregulation across interconnected physiological systems. The cumulative biological risk score (CBRS) used in the present study was designed as a focused indicator of dysregulation across hematologic, metabolic, anthropometric, inflammatory, and nutritional domains. Although the CBRS is not equivalent to a full allostatic load index, it captures the accumulation of selected biological abnormalities that may be relevant to hypertension in older adults. Anemia was included as a component of the CBRS because it may reflect hematologic vulnerability and reduced physiological reserve in older adults. In later life, anemia is frequently associated with nutritional deficiency, chronic inflammation, chronic kidney disease, and multimorbidity [10]. These conditions may coexist with metabolic and inflammatory dysregulation and may indicate accumulated biological vulnerability in older adults. Therefore, anemia was considered not merely as a hematologic condition but as a clinically meaningful biological risk indicator within the CBRS framework.

The rationale for integrating these indicators into a single score is that cumulative biological burden may arise from the coexistence of multiple modest abnormalities rather than from one severe abnormality alone [9]. In older adults, metabolic dysregulation, adiposity, inflammation, hematologic vulnerability, and nutritional deficiency frequently overlap and may interact through shared vascular and inflammatory pathways. A cumulative score approach therefore provides a simple way to summarize the number of dysregulated biological indicators and to classify individuals according to the degree of clustered biological vulnerability. This approach is consistent with allostatic load research, in which multiple biomarkers are commonly combined into a summary index to estimate cumulative physiological burden and predict adverse health outcomes [11]. From a research perspective, the CBRS allows examination of whether hypertension shows a graded association with the accumulation of biological and metabolic abnormalities, beyond the effects of individual risk factors.

From a biological perspective, several mechanisms may explain the association between cumulative biological risk and hypertension. Insulin resistance and adiposity can promote endothelial dysfunction, vascular inflammation, and increased arterial stiffness [4,12]. Vitamin D deficiency has been linked to blood pressure regulation through potential effects on the renin–angiotensin–aldosterone system and vascular function [13]. Magnesium also plays a role in vascular smooth muscle relaxation, calcium handling, endothelial function, and peripheral vascular resistance, all of which are relevant to blood pressure control [6,14].

From a nursing perspective, assessment of cumulative biological risk may provide a broader description of clustered biological vulnerabilities among older adults. In this context, an integrated assessment of biological risk may provide nurses with a broader understanding of an individual’s physiological risk profile. Such an approach may inform comprehensive nursing assessment, risk communication, and consideration of lifestyle-related factors, while further validation is required before clinical screening use.

Previous studies have shown that allostatic load and cumulative biological risk are associated with cardiovascular disease, morbidity, and mortality in aging populations [8,15]. These findings suggest that the accumulation of biological dysregulation across multiple systems may be relevant to cardiovascular vulnerability and blood pressure regulation. However, prior research has often focused on broad allostatic load indices or individual cardiometabolic biomarkers, and relatively little is known about whether a focused CBRS based on routinely available indicators is associated with hypertension among Korean older adults. Moreover, evidence using nationally representative Korean data with complex sample analysis remains limited. Therefore, this study aimed to examine the association between CBRS and physician-diagnosed hypertension among older adults using data from the Korea National Health and Nutrition Examination Survey.

## METHODS

### 1. Study design and data source

This study was a cross-sectional secondary analysis using data from the 2024 Korea National Health and Nutrition Examination Survey (KNHANES), which was publicly released in December 2025. This study was reported in accordance with the Strengthening the Reporting of Observational Studies in Epidemiology guideline for cross-sectional studies. KNHANES is a nationally representative surveillance system designed to assess health behaviors, chronic disease prevalence, and nutritional status in the Korean population using a complex, multistage, stratified cluster sampling design [16].

The survey employs a complex, multistage, stratified cluster sampling design based on the national census sampling frame, in which primary sampling units are selected first, followed by households as secondary sampling units. Stratification variables include region (province), urban–rural classification, and housing type, with additional implicit stratification factors such as age distribution and household characteristics.

Data are collected through standardized health interviews, self-administered questionnaires, physical examinations, and laboratory tests conducted by trained personnel using mobile examination centers and standardized protocols.

### 2. Participants

Among 6,997 individuals included in the 2024 KNHANES, 1,951 participants aged 65 years or older were initially identified. Participants with missing data on depressive symptoms assessed using the Patient Health Questionnaire-9 (PHQ-9) or serum magnesium levels were excluded first (n = 301). Additional participants with missing or invalid values in hypertension status, CBRS components, sociodemographic variables, or health behavior variables were further excluded (n = 99). Finally, 1,551 older adults were included in the main analytic sample. Valid sample sizes differed slightly across variables because of variable-specific missing or invalid values; diabetes status and aerobic physical activity in Table 1 included 1,550 and 1,546 participants, respectively. The final complex sample logistic regression model included 1,546 participants with complete data for all model variables. The participant selection process is presented in Appendix Figure 1.

### 3. Measures

#### 1) Outcome variable: Hypertension

Hypertension was defined as self-reported physician-diagnosed hypertension based on the KNHANES health interview data.

#### 2) Main exposure: CBRS

The CBRS was calculated by assigning 1 point for each risk indicator present and 0 points for each indicator absent, yielding a total score ranging from 0 to 5. The five indicators were anemia, elevated glycated hemoglobin (glycated hemoglobin [HbA1c] ≥ 5.7%), obesity defined as body mass index [BMI] ≥ 25 kg/m^2^, elevated high-sensitivity C-reactive protein [hs-CRP] ≥ 3 mg/L, and vitamin D deficiency defined as serum 25-hydroxyvitamin D < 20 ng/mL. The CBRS included hematologic, metabolic, anthropometric, inflammatory, and nutritional indicators. BMI was included as an anthropometric indicator of adiposity rather than as a biochemical marker. The HbA1c threshold of 5.7% was selected based on the American Diabetes Association criterion for prediabetes, which defines HbA1c levels of 5.7%–6.4% as prediabetes [17]. Anemia was defined as a hemoglobin concentration < 13.0 g/dL in men and < 12.0 g/dL in women, according to the World Health Organization guideline [18]. BMI ≥ 25 kg/m^2^ was used according to the Korean obesity criteria [19], and vitamin D deficiency was defined using the commonly applied threshold of serum 25-hydroxyvitamin D < 20 ng/mL [20].

For interpretability, participants were categorized a priori according to the number of accumulated biological risk indicators: low risk (0), moderate risk (1–2), and high risk (≥ 3). This categorization distinguished participants with no risk indicators from those with limited accumulation and those with multiple concurrent abnormalities. The grouping was conceptually informed by cumulative biological risk and allostatic load research, in which multiple dysregulated indicators are combined into a summary index [9,11]. However, these categories were defined for analytic interpretability and should not be regarded as clinically validated cutoff values. In this study, HbA1c ≥ 5.7% was used as a biological risk indicator of glycemic dysregulation within the CBRS, whereas diabetes status represented physician-diagnosed disease status. Diabetes status was included in the descriptive analysis but was not entered into the final multivariable model to avoid conceptual overlap with the HbA1c component of the CBRS.

#### 3) Covariates

Covariates included sex, age, household income, education level, smoking status, monthly alcohol use, aerobic physical activity, depressive symptoms, and serum magnesium levels. Household income was categorized into quartiles (Q1–Q4), and education level was categorized as elementary school or less, middle school, high school, and college or higher. Smoking status was classified as smoker or non-smoker. Monthly alcohol use was defined as drinking alcohol at least once per month, and aerobic physical activity was categorized as yes or no according to the KNHANES definition. These sociodemographic and health behavior variables were selected based on their known associations with hypertension and blood pressure regulation [1,21]. Depressive symptoms were assessed using the PHQ-9, which has been validated in the Korean population [22], and were included as a continuous variable based on previous evidence linking depression to hypertension risk [23]. Serum magnesium levels were treated as a continuous variable because of their relevance to vascular tone and blood pressure regulation [6]. Diabetes status was presented in the descriptive analysis to characterize clinical differences according to hypertension status; however, it was not included in the final multivariable model because elevated glycated hemoglobin, which reflects glycemic dysregulation, was already included as a component of the CBRS.

### 4. Statistical analysis

All analyses accounted for the complex sampling design of KNHANES by incorporating sampling weights, stratification variables, and cluster variables. Categorical variables were presented as unweighted frequencies and weighted percentages, whereas continuous variables were presented as weighted means and standard errors. Group differences were assessed using Rao–Scott adjusted F tests for categorical variables and complex sample general linear models for continuous variables.

Covariates were selected a priori based on previous evidence, biological plausibility, and their potential roles as confounders. Complex sample logistic regression analysis was performed with all covariates entered simultaneously using the enter method. Adjusted odds ratios and 95% confidence intervals (CIs) were calculated. Multicollinearity was assessed using tolerance and variance inflation factor values, and the linearity-in-the-logit assumption was examined for continuous covariates. No violations were identified.

All tests were two-sided, with *p* < .05 considered statistically significant. Analyses were conducted using IBM SPSS Statistics version 30.0 (IBM Corp., Armonk, NY, USA) with the Complex Samples module.

### 5. Ethical considerations

KNHANES is conducted in accordance with national regulations and was approved by the Institutional Review Board (IRB) of the Korea Disease Control and Prevention Agency (IRB No. 2022-11-16-R-03). All participants provided written informed consent at the time of the original survey. In accordance with the Declaration of Helsinki, participant anonymity and confidentiality were protected throughout the original data collection and public data release process. The dataset used in this study is publicly available and fully de-identified; therefore, the researchers had no access to personally identifiable information and no direct contact with participants. This secondary analysis was reviewed and approved as exempt by the IRB of Daejin University (IRB No. 1040656-202603-HR-01-13).

## RESULTS

### 1. General characteristics of participants according to hypertension status

The general characteristics of the participants according to hypertension status are presented in Table 1. Participants with hypertension were older than those without hypertension (73.23 ± 0.21 vs. 71.57 ± 0.23 years; F = 28.90, *p* < .001). There was no significant difference in sex distribution according to hypertension status (Rao–Scott F = 0.014, *p* = .905). Household income differed significantly by hypertension status (Rao–Scott F = 3.68, *p* = .013), with a higher weighted proportion of participants in the lowest income quartile among those with hypertension. Educational level also differed significantly between groups (Rao–Scott F = 3.32, *p* = .016), with a higher weighted proportion of participants with elementary education or less in the hypertension group.

Current smoking and monthly alcohol use were not significantly associated with hypertension status (Rao–Scott F = 3.10, *p* = .080; Rao–Scott F = 0.30, *p* = .586, respectively). Aerobic physical activity differed significantly by hypertension status (Rao–Scott F = 7.22, *p* = .008), with a higher weighted proportion of physically inactive participants among those with hypertension. Diabetes was also more prevalent among participants with hypertension than among those without hypertension (29.6% vs. 17.9%; Rao–Scott F = 12.41, *p* < .001). PHQ-9 score and serum magnesium level did not differ significantly according to hypertension status (F = 0.71, *p* = .411; F = 0.27, *p* = .601, respectively).

### 2. Distribution of biological risk factors and CBRS according to hypertension status

The distribution of biological risk factors and CBRS according to hypertension status is presented in Table 2. There were no significant differences in anemia (Rao–Scott F = 3.05, *p* = .083), elevated hs-CRP (Rao–Scott F = 3.19, *p* = .076), or vitamin D deficiency (Rao–Scott F = 3.22, *p* = .075) according to hypertension status. In contrast, the proportion of participants with HbA1c ≥ 5.7% was significantly higher among those with hypertension than among those without hypertension (65.0% vs. 52.1%; Rao–Scott F = 26.37, *p* < .001). Similarly, obesity defined as BMI ≥ 25 kg/m^2^ was more prevalent among participants with hypertension than among those without hypertension (46.3% vs. 24.5%; Rao–Scott F = 65.98, *p* < .001).

The distribution of CBRS categories differed significantly according to hypertension status (Rao–Scott F = 13.45, *p* < .001). The weighted proportion of participants in the high-risk group was higher among those with hypertension than among those without hypertension (25.3% vs. 15.0%), whereas the weighted proportion of participants in the low-risk group was lower among those with hypertension than among those without hypertension (9.3% vs. 15.9%).

### 3. Factors associated with hypertension

The results of the complex sample logistic regression analysis examining factors associated with hypertension are shown in Table 3. CBRS showed a graded association with hypertension across categories. Compared with the low-risk group, participants in the moderate-risk group had higher odds of hypertension (odds ratio [OR] = 1.67, 95% CI = 1.16–2.40, *p* = .006), and those in the high-risk group had even greater odds of hypertension (OR = 2.92, 95% CI = 1.87–4.54, *p* < .001).

Age was positively associated with hypertension (OR = 1.06, 95% CI = 1.04–1.09, *p* < .001). Monthly alcohol use was also associated with higher odds of hypertension (OR = 1.33, 95% CI = 1.03–1.72, *p* = .031). In addition, absence of aerobic physical activity was associated with higher odds of hypertension (OR = 1.42, 95% CI = 1.08–1.87, *p* = .013). Sex, household income, education level, current smoking, PHQ-9 score, and serum magnesium level were not significantly associated with hypertension in the adjusted model. The overall model was statistically significant (Wald F = 5.04, *p* < .001), with a Nagelkerke pseudo R^2^ of .08 as a model fit index.

## DISCUSSION

This study examined the association between cumulative biological risk and hypertension among older adults in Korea using nationally representative survey data. The main finding was that CBRS showed a graded association with hypertension; compared with the low-risk group, the moderate- and high-risk groups had progressively higher odds of hypertension. This graded pattern suggests that the accumulation of biological and metabolic abnormalities may be relevant to hypertension in later life.

The graded association across CBRS categories is a key finding of this study. However, this finding should be interpreted cautiously. In Table 2, elevated HbA1c and obesity differed significantly according to hypertension status, whereas anemia, elevated hs-CRP, and vitamin D deficiency did not. Therefore, the observed association between CBRS and hypertension may have been influenced substantially by glycemic dysregulation and obesity rather than reflecting equal contributions from all five components. Accordingly, the CBRS should be interpreted as a composite indicator summarizing clustered biological abnormalities. The present findings do not establish that the CBRS is more informative than individual risk factors or that each component contributes independently to hypertension.

The potential clinical relevance of the CBRS lies in its ability to summarize several biological abnormalities into a single descriptive profile. In nursing practice, this concept may facilitate discussion of clustered biological vulnerability and encourage comprehensive assessment of metabolic, inflammatory, nutritional, and lifestyle-related factors. However, the CBRS has not been validated as a clinical screening or prediction tool. Its sensitivity, specificity, discrimination, calibration, and optimal cutoff were not evaluated in this study. Therefore, the present findings support the CBRS only as a screening-oriented research concept and not as a tool for clinical decision-making.

These findings are consistent with the concepts of cumulative biological risk and allostatic load. Allostatic load refers to multisystem physiological dysregulation that develops through repeated or chronic biological stress exposure [9]. Previous studies have described allostatic load as a framework for assessing cumulative biological risk across physiological systems [11] and have linked higher allostatic load to morbidity, mortality, and cardiovascular disease risk in older adults [8,15]. In this context, the CBRS used in the present study can be interpreted as a focused biological risk indicator of cumulative physiological burden. Although it is not identical to a full allostatic load index, it reflects the same conceptual direction: multiple biological disturbances may have greater clinical meaning when considered together.

The components of the CBRS should be interpreted as part of an integrated biological risk profile rather than as isolated predictors. Elevated HbA1c in this study reflects glycemic dysregulation, including prediabetic-range abnormality, rather than directly measured insulin resistance. Glycemic dysregulation may contribute to hypertension through vascular dysfunction, impaired metabolic regulation, endothelial injury, and increased arterial stiffness [4]. Although insulin resistance was not directly measured in this study, previous research suggests that metabolic and vascular insulin resistance are involved in cardiovascular disease mechanisms [24], and population-based evidence has linked insulin resistance-related metabolic scores to hypertension in middle-aged and older adults [25]. Therefore, the HbA1c component of the CBRS should be interpreted cautiously as an indicator of glycemic dysregulation, not as a direct measure of insulin resistance.

Obesity within the CBRS should also be understood as more than excess body weight. In older adults, adiposity is associated with inflammatory biomarker changes and cardiometabolic vulnerability [5]. Obesity-related adipose tissue dysfunction may promote chronic inflammation, oxidative stress, altered adipokine secretion, and vascular injury [26]. Recent evidence further suggests that obesity-driven vascular dysfunction involves endothelial injury, arterial stiffening, and microvascular changes [27]. Thus, obesity may contribute to hypertension as part of a broader metabolic–inflammatory risk pattern rather than as an isolated anthropometric factor.

Inflammation is another important component of the integrated risk profile. Although elevated hs-CRP was not individually significant in the group comparison in this study, it remains biologically meaningful as one component of cumulative risk. A recent systematic review and meta-analysis reported an association between C-reactive protein and 24-hour blood pressure, particularly at CRP levels of 3 mg/L or higher [28]. High-sensitivity C-reactive protein has also been linked to age-related vascular dysfunction [29] and longitudinal changes in arteriosclerosis [30]. These findings support the interpretation that inflammatory burden may contribute to hypertension not necessarily as a single independent factor, but as part of accumulated biological dysregulation.

Vitamin D deficiency was included in the CBRS because vitamin D may be relevant to vascular function and blood pressure regulation. A recent systematic review described the potential role of vitamin D in blood pressure regulation, although the effects of supplementation on blood pressure remain inconsistent [7]. A 2024 review also noted that the relationship between vitamin D and hypertension is complex and may vary depending on clinical context [13]. Therefore, the present findings should not be interpreted as evidence that vitamin D deficiency alone causes hypertension. Rather, vitamin D deficiency may be considered one component of an unfavorable biological risk profile.

Serum magnesium was included as a covariate rather than a component of the CBRS. In this study, serum magnesium was not independently associated with hypertension in the adjusted model. This null finding should be interpreted cautiously because serum magnesium may not fully represent total body magnesium status. Magnesium is physiologically relevant to vascular tone, endothelial function, calcium handling, and vascular smooth muscle relaxation [6,31]. An umbrella meta-analysis also reported that magnesium supplementation was associated with reductions in systolic and diastolic blood pressure under certain dose and duration conditions [32]. However, because the present study did not examine magnesium supplementation or intracellular magnesium status, this finding should be interpreted only as an adjusted association involving serum magnesium.

The finding that absence of aerobic physical activity was associated with higher odds of hypertension is consistent with the biological and clinical relevance of physical activity in vascular health. Physical inactivity has been associated with poorer endothelial function [33]. Exercise interventions have been reported to improve endothelial function in patients with hypertension [34]. In older adults with hypertension, aerobic exercise has also been shown to support cardiovascular health management [35]. In the context of cumulative biological risk, physical activity may be important not only as a behavioral factor directly related to blood pressure regulation but also as a modifiable factor that can influence obesity, glycemic status, inflammation, and overall biological risk accumulation.

Monthly alcohol use was associated with higher odds of hypertension in the adjusted model. This finding should be interpreted cautiously because monthly alcohol use in KNHANES reflects drinking frequency rather than detailed alcohol dose, drinking pattern, or lifetime drinking history. A dose–response meta-analysis of cohort studies reported that alcohol intake is generally associated with higher blood pressure levels [21]. Therefore, the present finding may reflect the adverse association between alcohol consumption and blood pressure regulation, although residual confounding, differences in drinking amount, drinking pattern, medication-related behavior change, and reverse causation cannot be excluded in this cross-sectional study.

From a biological nursing perspective, these findings support the need for comprehensive risk assessment and preventive management among older adults. Rather than focusing only on a single laboratory abnormality, nurses may consider the accumulation of biological and metabolic abnormalities as an indicator of clustered biological vulnerability. This approach may provide a conceptual basis for recognizing clustered biological vulnerabilities in older adults. However, decisions regarding blood pressure monitoring, referral, or intervention should continue to be based on established clinical assessments and guidelines. Preventive nursing strategies may include physical activity promotion, weight management, metabolic monitoring, nutritional assessment, and education regarding inflammation- and vascular health-related risk factors. Such an approach is particularly relevant for community-dwelling older adults, in whom hypertension often coexists with metabolic, inflammatory, and nutritional vulnerabilities.

Several limitations should be considered. First, the cross-sectional design prevents causal inference, and reverse causation cannot be excluded. Second, hypertension was defined based on self-reported physician diagnosis, which may underestimate undiagnosed hypertension. Third, the CBRS and its risk categories were constructed using predefined cutoff values for interpretability and have not been clinically validated. This approach may not fully capture the continuous nature of biological risk. Fourth, each CBRS component was assigned an equal weight despite potentially differing contributions to hypertension-related risk. Component-specific, leave-one-component-out, continuous-score, and model-comparison analyses were not performed. Because elevated HbA1c and obesity were the only components that differed significantly by hypertension status, the observed association may have been influenced primarily by these components rather than reflecting an independent cumulative effect across all five indicators. Fifth, potentially relevant factors, including antihypertensive medication use, dietary sodium intake, kidney function, and detailed alcohol consumption patterns, were not fully considered, and residual confounding may remain. Sixth, the CBRS was not developed or validated as a clinical screening or prediction tool, and its discrimination, calibration, sensitivity, specificity, and optimal cutoff values were not evaluated. Therefore, the findings should not be used to guide individual clinical decisions. Future studies should examine the individual and combined contributions of CBRS components, clarify temporal relationships, and evaluate the predictive and clinical utility of the CBRS.

## CONCLUSION

Higher CBRS categories were associated with higher odds of hypertension among Korean older adults. However, because elevated HbA1c and obesity were the only components that differed significantly by hypertension status, the observed association may have been influenced primarily by these components. Therefore, the findings indicate an association with the composite score but do not establish an independent cumulative effect across all five indicators. The CBRS may provide a research framework for describing clustered biological vulnerability but should not be regarded as a validated clinical screening or prediction tool. Further component-specific, sensitivity, and longitudinal analyses are needed to clarify the contribution of each indicator and evaluate the clinical utility of the CBRS.

## Figures and Tables

**Table 1.** General Characteristics of Participants According to Hypertension Status (N = 1,551)

Variables	n (%) or M ± SE	Without hypertension n (%)	With hypertension n (%)	Rao–Scott F or F	*p*
Age (years)	72.46 ± 0.17	71.57 ± 0.23	73.23 ± 0.21	28.90	<.001
Sex				0.014	.905
Women	886 (53.3)	406 (53.5)	480 (53.2)		
Men	665 (46.7)	313 (46.5)	352 (46.8)		
Household income				3.68	.013
Q1 (lowest)	589 (35.1)	242 (30.9)	347 (38.7)		
Q2	480 (32.2)	237 (34.0)	243 (30.5)		
Q3	317 (21.3)	163 (23.6)	154 (19.4)		
Q4 (highest)	165 (11.4)	77 (11.5)	88 (11.4)		
Education level				3.32	.016
Elementary school or less	675 (41.3)	285 (37.7)	390 (44.4)		
Middle school	330 (21.4)	159 (22.4)	171 (20.5)		
High school	359 (24.1)	180 (25.6)	179 (22.9)		
College or higher	187 (13.2)	95 (14.3)	92 (12.2)		
Current smoking				3.10	.080
No	1,422 (91.6)	652 (90.1)	770 (92.9)		
Yes	129 (8.4)	67 (9.9)	62 (7.1)		
Monthly alcohol use				0.30	.586
No	1,018 (64.1)	478 (64.9)	540 (63.4)		
Yes	533 (35.9)	241 (35.1)	292 (36.6)		
Aerobic physical activity				7.22	.008
No	1,061 (67.2)	463 (63.6)	598 (70.4)		
Yes	485 (32.8)	253 (36.4)	232 (29.6)		
Diabetes				12.41	<.001
No	1,178 (75.9)	590 (82.1)	588 (70.4)		
Yes	372 (24.1)	129 (17.9)	243 (29.6)		
PHQ-9 score	1.51 ± 0.09	1.43 ± 0.11	1.57 ± 0.12	0.71	.411
Serum magnesium, mg/dL	2.13 ± 0.01	2.14 ± 0.01	2.13 ± 0.01	0.27	.601

Categorical variables are presented as unweighted frequencies (n) and weighted percentages (%), and continuous variables are presented as weighted means and standard errors. *p*-values were obtained using Rao–Scott adjusted F tests for categorical variables and complex sample general linear models for continuous variables. All analyses accounted for the complex survey design, including sampling weights, stratification variables, and cluster variables. Valid sample sizes differed slightly across variables because missing or invalid codes were excluded from each analysis. M = Mean; SE = Standard error; PHQ-9 = Patient Health Questionnaire-9.

**Table 2.** Distribution of Biological Risk Indicators and Cumulative Biological Risk Score According to Hypertension Status Using Complex Sample Analysis (N = 1,551)

Variables	Without hypertension n (%)	With hypertension n (%)	Rao–Scott F	*p*
Anemia			3.05	.083
No	628 (88.1)	704 (84.9)		
Yes	91 (11.9)	128 (15.1)		
HbA1c ≥ 5.7%			26.37	< .001
No	346 (47.9)	290 (35.0)		
Yes	373 (52.1)	542 (65.0)		
BMI ≥ 25 kg/m^2^			65.98	< .001
No	536 (75.5)	444 (53.7)		
Yes	183 (24.5)	388 (46.3)		
hs-CRP ≥ 3 mg/L			3.19	.076
No	650 (89.3)	769 (92.2)		
Yes	69 (10.7)	63 (7.8)		
Vitamin D < 20 ng/mL			3.22	.075
No	387 (54.4)	413 (49.6)		
Yes	332 (45.6)	419 (50.4)		
CBRS group			13.45	< .001
Low (0)	117 (15.9)	75 (9.3)		
Moderate (1–2)	496 (69.1)	547 (65.4)		
High (≥ 3)	106 (15.0)	210 (25.3)		

Values are presented as unweighted frequencies (n) and weighted percentages (%). *p*-values were obtained using Rao–Scott adjusted F tests for complex sample analysis. All analyses accounted for the complex survey design, including sampling weights, stratification variables, and cluster variables. CBRS was calculated as the sum of the following five components: anemia, HbA1c ≥ 5.7%, BMI ≥ 25 kg/m^2^, hs-CRP ≥ 3 mg/L, and vitamin D < 20 ng/mL. HbA1c = Glycated hemoglobin; BMI = Body mass index; hs-CRP = High-sensitivity C-reactive protein; CBRS = Cumulative biological risk score.

**Table 3.** Factors Associated with Hypertension in Complex Sample Logistic Regression (N = 1,546)

Variables	B	SE	OR	95% CI	*p*
CBRS group					
Low (0)	Ref.		1.00		
Moderate (1–2)	0.51	0.18	1.67	1.16–2.40	.006
High (≥ 3)	1.07	0.22	2.92	1.87–4.54	< .001
Sex					
Women	Ref.		1.00		
Men	−0.01	0.13	0.99	0.76–1.29	.956
Age (years)	0.06	0.01	1.06	1.04–1.09	< .001
Household income					
Q4, highest	Ref.		1.00		
Q3	−0.25	0.21	0.78	0.52–1.17	.222
Q2	−0.22	0.18	0.80	0.56–1.16	.239
Q1, lowest	−0.05	0.21	0.95	0.63–1.43	.797
Education level					
College or higher	Ref.		1.00		
High school	0.02	0.20	1.02	0.68–1.52	.937
Middle school	0.02	0.23	1.02	0.64–1.56	.943
Elementary school or less	0.10	0.21	1.11	0.72–1.69	.642
Current smoking					
No	Ref.		1.00		
Yes	−0.34	0.22	0.71	0.47–1.09	.116
Monthly alcohol use					
No	Ref.		1.00		
Yes	0.28	0.13	1.33	1.03–1.72	.031
Aerobic physical activity					
Yes	Ref.		1.00		
No	0.35	0.14	1.42	1.08–1.87	.013
PHQ-9 score	0.02	0.02	1.02	0.98–1.06	.342
Serum magnesium, mg/dL	−0.18	0.36	0.83	0.41–1.69	.609

Reference categories were the low-risk CBRS group, women, household income Q4, college or higher education, no current smoking, no monthly alcohol use, and participation in aerobic physical activity. All covariates were entered simultaneously into the model. Analyses accounted for the complex sampling design, including sampling weights, stratification variables, and cluster variables. The final model included 1,546 participants with complete data for all model variables. Diagnostic testing indicated no problematic multicollinearity or violation of the linearity-in-the-logit assumption for continuous covariates. Nagelkerke R^2^ = .08 and is reported as a pseudo-R^2^ index of model fit. B = Regression coefficient; SE = Standard error; OR = Odds ratio; CI = Confidence interval; CBRS = Cumulative biological risk score; PHQ-9 = Patient Health Questionnaire-9.
